# Risedronate to Prevent Bone Loss After Sleeve Gastrectomy: Study Design and Feasibility Report of a Pilot Randomized Controlled Trial

**DOI:** 10.1002/jbm4.10407

**Published:** 2020-10-02

**Authors:** Ashlyn A Swafford, Jamy D Ard, Daniel P Beavers, Peri C Gearren, Adolfo Z Fernandez, Sherri A Ford, Katelyn A Greene, Daniel E Kammire, Beverly A Nesbit, Kylie K Reed, Ashley A Weaver, Kristen M Beavers

**Affiliations:** ^1^ Deparment of Health and Exercise Science Wake Forest University Winston‐Salem NC USA; ^2^ Weight Management Center Wake Forest Baptist Medical Center Winston‐Salem NC USA; ^3^ Department of Biostatistics and Data Science Wake Forest School of Medicine Winston‐Salem NC USA; ^4^ Department of Biomedical Engineering Wake Forest School of Medicine Winston‐Salem NC USA

**Keywords:** DXA, ANTIRESORPTIVES, FRACTURE PREVENTION, CLINICAL TRIALS, BONE QUANTITATIVE COMPUTED TOMOGRAPHY

## Abstract

Mounting evidence implicates bariatric surgery as a cause of increased skeletal fragility and fracture risk. Bisphosphonate therapy reduces osteoporotic fracture risk and may be effective in minimizing bone loss associated with bariatric surgery. The main objective of this pilot randomized controlled trial (RCT; Clinical Trial No. NCT03411902) was to determine the feasibility of recruiting, treating, and following 24 older patients who had undergone sleeve gastrectomy in a 6 month RCT examining the efficacy of 150‐mg once‐monthly risedronate (versus placebo) in the prevention of surgical weight‐loss–associated bone loss. Feasibility was defined as: (i) >30% recruitment yield, (ii) >80% retention, (iii) >80% pills taken, (iv) <20% adverse events (AEs), and (v) >80% participant satisfaction. Study recruitment occurred over 17 months. Seventy participants were referred, with 24 randomized (34% yield) to risedronate (*n* = 11) or placebo (*n* = 13). Average age was 56 ± 7 years, 83% were female (63% postmenopausal), and 21% were black. The risedronate group had a higher baseline BMI than the placebo group (48.1 ± 7.2 versus 41.9 ± 3.8 kg/m^2^). The 10‐year fracture risk was low (6.0% major osteoporotic fracture, 0.4% hip fracture); however, three individuals (12.5%, all risedronate group) were osteopenic at baseline. Twenty‐one participants returned for 6‐month follow‐up testing (88% retention) with all (*n* = 3) loss to follow‐up occurring in the risedronate group. Average number of pills taken among completers was 5.9 ± 0.4 and 6.0 ± 0.0 in the risedronate and placebo groups, respectively (*p* = 0.21), with active participants taking >80% of allotted pills. Five AEs (3.7% AE rate) were reported; one definitely related, four not related, and none serious. All participants reported high satisfaction with participation in the study. Use of bisphosphonates as a novel therapeutic to preserve bone density in patients who had undergone a sleeve gastrectomy appears feasible and well‐tolerated. Knowledge gained from this pilot RCT will be used to inform the design of an appropriately powered trial.

**Clinical Trial Registration:**

http://clinicaltrials.gov/show/NCT03411902. Weight Loss With Risedronate for Bone Health. © 2020 The Authors. *JBMR Plus* published by Wiley Periodicals LLC on behalf of American Society for Bone and Mineral Research.

## Introduction

Despite well‐recognized improvements in body weight and cardiometabolic indices, mounting evidence implicates bariatric surgery as a cause of increased skeletal fragility and fracture risk. As of 2018, approximately 252,000 bariatric surgeries were performed in the United States, with the increasingly popular sleeve gastrectomy (SG) procedure comprising 61% of all surgical types.^(^ ^1^ ^)^ Prospective data consistently report hip BMD losses of 3% to 7% in the 6 to 12 months following SG^(^ ^2^, ^3^ ^)^—similar to other bariatric procedures^(^ ^4^ ^)^— that appear to persist after the cessation of weight loss.^(^ ^5^ ^)^ Data also link mixed bariatric surgical procedures with reductions in bone quality,^(^ ^6^, ^7^ ^)^ although less is known about SG, specifically. Importantly, newly emerging data show bariatric surgery increases the risk of overall fracture by 20% to 200%,^(^ ^8^, ^9^ ^)^ including SG.^(^ ^10^, ^11^ ^)^ Taken together, increased fracture risk is a growing concern of both bariatric surgeons and their patients, providing impetus for the identification of effective strategies to minimize bone loss in this population.

Bisphosphonate therapy reduces osteoporotic fracture risk,^(^ ^12^ ^)^ and may be effective in minimizing bone loss associated with surgical weight loss. Once‐monthly oral risedronate is a commonly prescribed bisphosphonate with a favorable gastrointestinal profile.^(^ ^13^ ^)^ It acts by inhibiting the activity of osteoclast cells, thereby decreasing the rate of bone resorption.^(^ ^14^ ^)^ Because weight loss is associated with significantly increased bone resorption,^(^ ^15^ ^)^ bisphosphonate use may counter bone loss during active weight loss, thereby reducing long‐term fracture risk in SG patients. Current clinical practice guidelines support the consideration of oral bisphosphonate use in bariatric surgery patients with osteoporosis, provided concerns regarding absorption or potential anastomotic ulcerations are obviated;^(^ ^16^ ^)^ however, no published studies have examined whether bisphosphonates can prophylactically attenuate surgical weight‐loss–associated reductions in bone density and quality.

To begin to fill this knowledge gap, the main objective of the pilot WE RISE (Weight Loss With Risedronate for Bone Health) randomized controlled trial (RCT) was to determine the feasibility of recruiting, treating, and following 24 older SG patients into a 6‐month RCT examining the efficacy of risedronate use (versus placebo) in the prevention of surgical weight‐loss–associated loss of bone mass and quality. Herein, we report full study design details, as well as feasibility data, including: (i) participant recruitment and retention rates, (ii) adherence to and safety of a once‐monthly oral dose of risedronate or placebo, and (iii) participant satisfaction. Data will be used to aid in the design of an appropriately powered trial.

## Participants and Methods

### Patient population

Patients 40 years of age or older who were scheduled to have a SG were recruited from the Wake Forest Baptist Health Weight Management Clinic in Winston Salem, North Carolina. Patients had to meet standard criteria for bariatric surgery, including a BMI ≥40 kg/m^2^, or a BMI ≥35 kg/m^2^ with associated complications of obesity such as poorly controlled type 2 diabetes mellitus or obstructive sleep apnea, in addition to being medically cleared as safe for surgery with normal electrolytes, mineral and vitamin levels, and blood counts.^(^ ^17^ ^)^

Patients adhered to the following clinic visit schedule postsurgery: one overnight hospital stay; 30‐day nutrition and surgeon follow‐up; 3‐month nutrition and blood‐draw follow‐up; 6‐month surgeon, resting metabolic rate, and exercise follow‐up; 9‐month nutrition follow‐up; and 12‐month surgeon and resting metabolic rate follow‐up. Participants were asked to establish an exercise routine preoperatively that was done for at least 30 minutes, 3‐to‐5 days per week, with exercise recommendations postsurgery including daily walking and strength training beginning after their 30‐day follow‐up visit. The American Society of Metabolic and Bariatric Surgery (ASMBS) recommendations for the perioperative nutrition, metabolic, and nonsurgical support of patients who had had bariatric surgery were followed.^(^ ^16^ ^)^ Briefly, patients were recommended to consume (typically in the form of a multivitamin, though one was not provided) at least 3000 IU/d of vitamin D (if serum levels were below 30 ng/mL), 1200 to 1500 mg/d of calcium, and 90 to 120 mg/d of vitamin K. Protein recommendations were based on height and sex, ranging from 65 to 110 g/d: slightly higher than the ASMBS recommended intake of 46 g/d for women and 56 g/d for men.

### Eligibility and recruitment

All potential participants were referred by clinic staff after meeting medical, nutritional, and psychological presurgical requirements for SG. Once the potential participant's surgery was scheduled, study staff approached the patient for possible participation in the study. Full inclusion/exclusion criteria are presented in Table 1. Briefly, phase I exclusion criteria were evaluated by phone screen and included: scheduled SG surgery, age <40 or >79 years, baseline weight >450 lbs (204 kg; DXA scanner limit), chronic antireflux treatment, history of medical disorders known to affect bone metabolism, use of bone‐active medications, or a known allergy to risedronate. Patients who were given phase I clearance were further evaluated by the study physician, who reviewed medical examinations and clinical laboratory tests (eg, normal serum calcium or absence of significant renal dysfunction: estimated glomerular filtration rate [eGFR] <30 mL/min per 1.73 m^2^) to identify any health concerns that would preclude participants from safely participating in the study and achieving phase II clearance. Normal vitamin D status was not a criterion for entry into the study because normal vitamin D status (>20 ng/mL) or supplementation was required for surgical clearance. Eligible and interested participants were then referred to the study coordinator to read and sign an institutional review board‐approved informed consent form prior to enrollment.

**Table 1:** Study Inclusion and Exclusion Criteria

Clearance	Criteria	Inclusion	Exclusion	Assessment
Phase I	Sleeve gastrectomy	Yes		Referred from WMC
	Age	40–79 y		Self‐report
	Weight status		Weight >450 lbs (204 kg) (DXA limit)	scale
	Medication use		Regular use of growth hormones, oral steroids, or prescription osteoporosis medications; known allergies to bisphosphonates. Unstable gastric reflux requiring 2 or more additional doses per month of antireflux medication.	Medical record
	Research participation	Willing to provide informed consent; agree to all study procedures and assessments.	Current participation in other research study; unable to provide own transportation to study visits; unable to position on DXA scanner independently.	Self‐report
Phase II	Physician clearance	Study physician approves safe participation.	Participant presents with clinical contraindications (ie, eGFR <30 mL/min per 1.73 m^2^, hypocalcemia, osteoporosis, pregnancy, esophageal abnormalities, increased risk of ulceration or electrolyte abnormalities).	Medical record or study baseline DXA scan

eGFR = Estimated glomerular filtration rate; WMC = weight management clinic.

### Study design and randomization

This pilot double‐blinded RCT (http://clinicaltrials.gov/show/NCT03411902) involved 24 participants assigned (via computer‐generated block randomization, with stratification by sex) to take six doses of once‐monthly risedronate or placebo capsules over a 6‐month period. Official study assessments occurred at baseline and at 6 months, with an optional 12‐month assessment (monetary remuneration was given after the 6‐month assessment or at the 12‐month assessment if the patient chose to return).

**Fig 1:**
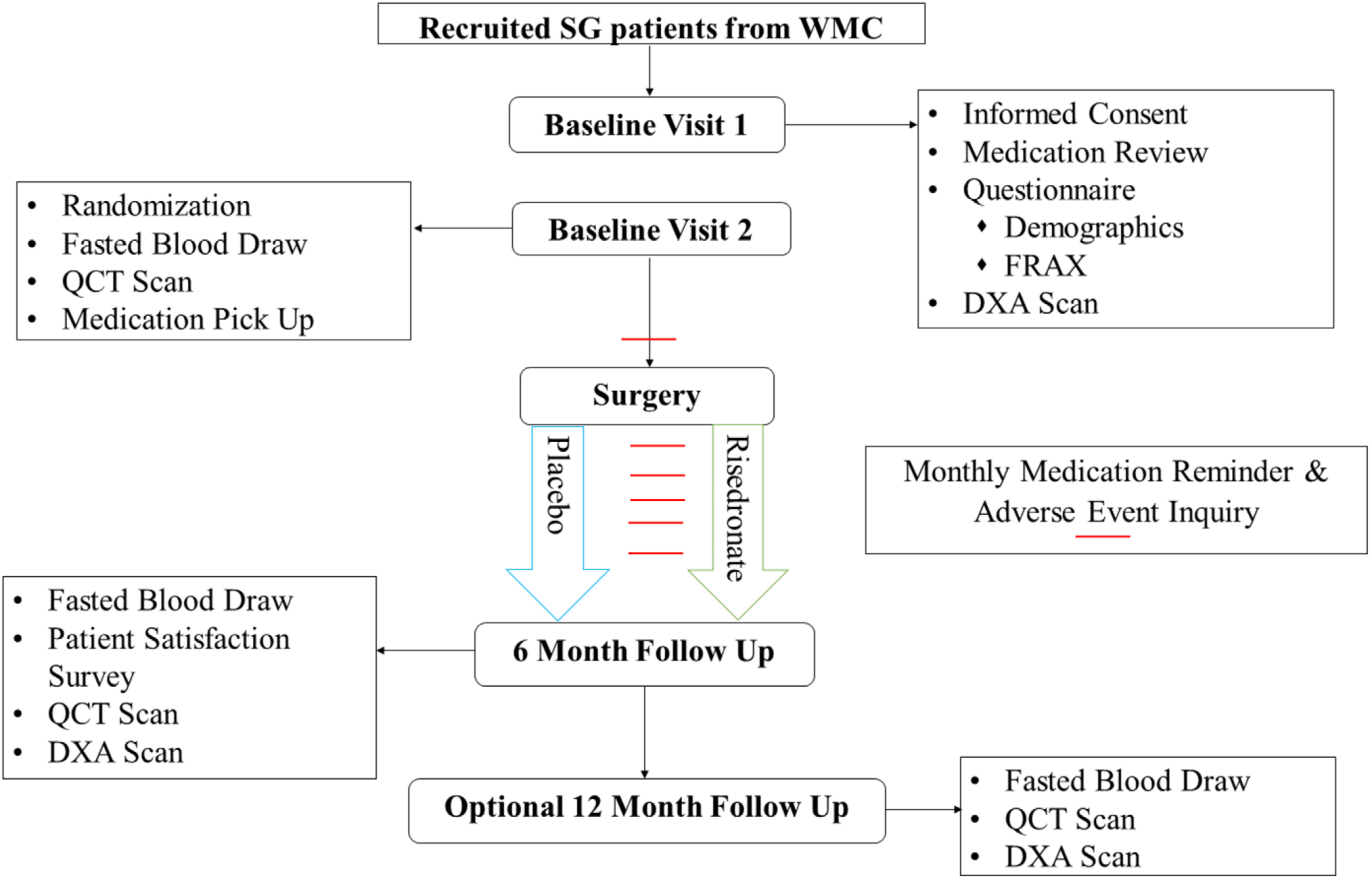
Weight Loss With Risedronate for Bone Health study flow diagram. FRAX = Fracture risk assessment tool; SG = sleeve gastrectomy; WMC = Weight Management Center.

An overview of the study timeline is provided in Fig. 1. Briefly, two in‐person baseline assessment visits occurred no more than 6 weeks prior to surgery and at least 3 days prior to surgery. At the first baseline assessment visit after providing informed consent and completing remuneration paperwork, participants were queried on self‐reported medical history and demographic characteristics, including age, race, postmenopausal status, education, and fracture history. The FRAX (fracture risk assessment tool; version 4.1)^(^ ^18^ ^)^ questionnaire and the first series of DXA scans (total body, hip, lumbar spine, and distal radius) were also completed. If osteoporosis (regional *T*‐score <2.5) was detected on any scan during the first baseline visit, participants were deemed ineligible and referred to their primary care physician.

At the second baseline visit, a fasted blood draw was performed along with a QCT scan of the lumbar spine and hip region (full scanning details presented below). At the end of this visit, participants were randomized to either risedronate or placebo groups, provided with their assigned medication, and instructed to consume the first dose 3 to 7 days prior to their surgery and monthly thereafter for the next 6 months. On each monthly medication date, participants were contacted by the study coordinator via phone to inquire if the pill had been taken that month and if any adverse events had occurred. Adverse events were classified as mild, moderate, severe, life‐threatening/disabling, or fatal and as not related, possibly related, or definitely related.

After 6 months, two in‐person follow‐up assessment visits occurred within 1 month from last medication dose (ie, 6‐month postsurgical date). As with baseline assessment visits, DXA scans, QCT scans, and a fasted blood draw occurred. Additionally, participants were asked to return their pill bottle, update their medication log, and complete a participant satisfaction survey containing nine Likert questions (1 = highly disagree, 5 = highly agree) regarding satisfaction with the study (see Supplementary Fig. SS1). Participants reported on overall communication, the medication process, if they found the study to be troublesome, study duration, and the blood‐draw process. The question, “Overall I was satisfied with my participation in this study” was used as the primary metric of participant satisfaction. Finally, for those who consented to 12‐month follow‐up testing, participants were asked to return after 6 months of free‐living conditions to complete additional in‐person follow‐up assessment visits occurring within a month and a half of their 1‐year postsurgical date. As with 6‐month assessments, DXA scans, QCT scans, and a fasted blood draw were performed.

### Outcome measures

The primary outcome for this pilot RCT is 6‐month feasibility defined as: (i) participant recruitment and retention rates, (ii) adherence to and safety of a once‐monthly oral dose of risedronate or placebo, and (iii) participant satisfaction. Secondary outcomes include a 6‐ and 12‐month change in: (i) DXA‐acquired hip, femoral neck, lumbar spine, and distal radius areal BMD (aBMD) and trabecular bone score (TBS) of the lumbar spine, and total body fat and lean masses; (ii) QCT acquired total hip and spine integral, cortical, and trabecular volumetric BMD (vBMD); and (iii) biomarkers of bone turnover, including P1NP and CTX. Details surrounding acquisition of the specific assessments are provided below. For completeness, all study outcome procedures are also described below; however, only feasibility outcome data are presented.

#### Feasibility metrics

To determine feasibility, this study assessed the retention and recruitment of 24 participants, determined the adherence to and safety of taking a monthly oral dose of 150‐mg risedronate, and evaluated the overall satisfaction of participants at 6 months postsurgery. Criteria for success were determined based upon prescribing information, best practices, and data from previously published trials^(^ ^19^, ^20^, ^21^, ^22^ ^)^ and included: (i) recruiting all 24 participants with a recruitment rate >30%; (ii) retaining >80% of the study sample, defined as completion of the 6‐month follow up visit; (iii) >80% of pill dosages taken (based on participant monthly self‐report and confirmed by 6‐month pill count); (iv) <20% total adverse events reported out of total number of contacts (conservatively assessed over 6 months, when possible); and (v) >80% of participants satisfied with participation, defined by the self‐reported Likert scale question, “Overall, I was satisfied with my participation in this study.” Based on concerns regarding hypocalcemia among patients taking bisphosphonates who had undergone bariatric surgery,^(^ ^23^ ^)^ posttreatment serum calcium data were abstracted from routine clinical laboratory panels assessed at 3 to 4 months.

#### DXA‐acquired body composition and bone metrics

All DXA‐acquired outcome measures were assessed at baseline, and at 6 and 12 months. If obtainable, total body composition and aBMD of the total hip, femoral neck, lumbar spine, and distal radius, as well as the TBS of the lumbar spine were determined by DXA (iDXA; GE Medical Systems, Madison, WI, USA). All scans were performed and analyzed in accordance with national recommendations by an ISCD‐ (International Society for Clinical Densitometry‐) trained DXA technologist, as done previously.^(^ ^24^ ^)^ Coefficients of variation from repeated measurements (on the same individual by the same technician) at our institution are <2% for total hip, femoral neck, and lumbar spine aBMD. In accordance with the ISCD standards, if a participant exceeded the field of view, the protocol was to acquire a full‐view scan of the right side of the body; the software mirrored that to compensate for the out‐of‐view left side.^(^ ^25^ ^)^

#### QCT‐acquired bone metrics

**Fig 2:**
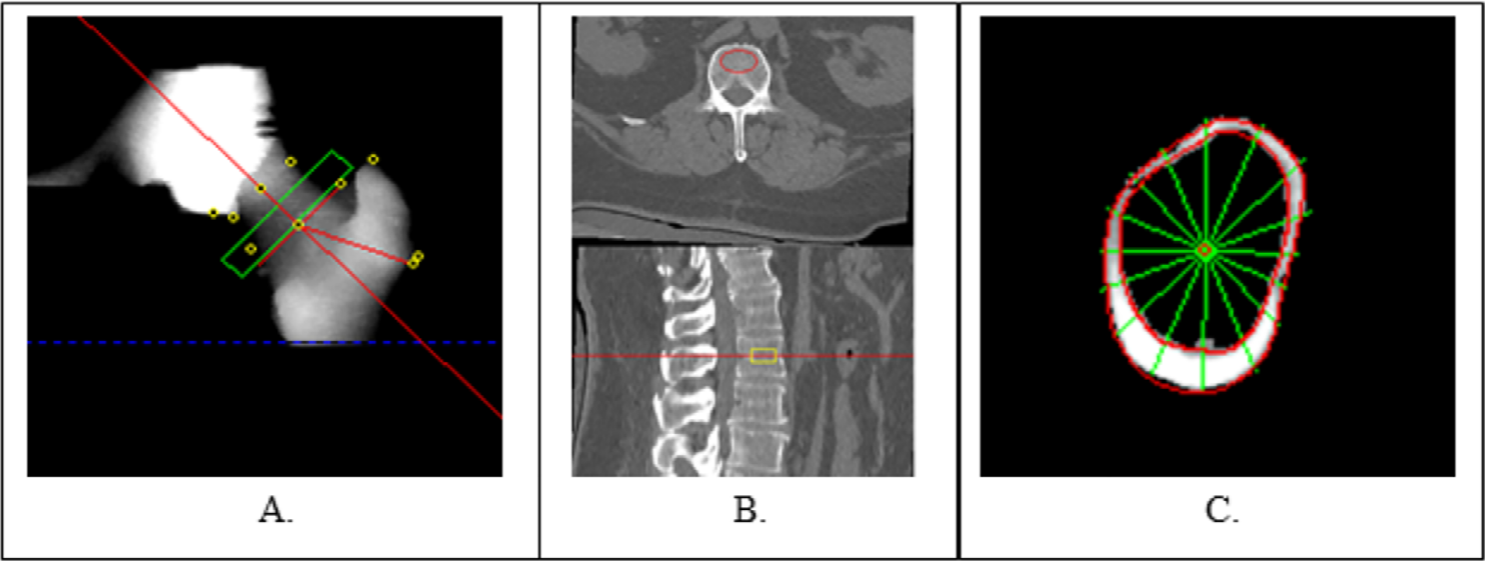
(*A*) Exemplar hip volumetric BMD analysis using computed tomography X‐ray absorptiometry hip module. (*B*) Lumbar spine analysis of volumetric BMD using three‐dimensional spine module. (*C*) Cortical thickness analysis of femoral neck using the Bone Investigational Toolkit software (Mindways Software, Austin, TX, USA).

All QCT‐acquired outcome measures were assessed at baseline, and 6 and 12 months. Helical CT scans of the hips and lumbar spine were acquired on a Siemens SOMATOM Definition Flash CT scanner (Siemens Healthineers, Erlangen, Germany) at Wake Forest Baptist Medical Center. The lumbar spine scan covered the region from the top of L1 through the base of L5; the bilateral hip scan covered the region from the superior acetabulum to midfemur. Both scans were conducted at a table height of 175 mm, 500‐mm scan field of view, 120 kV, 350 mA, 1‐mm helical mode with a pitch of 1, and a 0.8‐second gantry rotation speed, standard reconstruction, with secondary reconstruction using a bone algorithm specifying a 0.625‐mm‐slice thickness. Lumbar spine scans also included a secondary reconstruction with 2‐mm slice thickness using an iterative metal reduction algorithm. A five‐port bone mineral calibration (as shown in Supplementary Fig. SS2) phantom (Mindways Software, Austin, TX, USA) was imaged in every scan to allow for measurement of vBMD. Trabecular vBMD was measured at the L1 to L4 levels; trabecular, cortical, and integral vBMD was measured for the right total hip, trochanter, and femoral neck using the QCT Pro three‐dimensional spine module (version 6.1) and the computed tomography X‐ray absorptiometry (CTXA) hip module (Mindways Software, Austin, TX, USA; Fig. 2*A*,*B*). Cortical thickness was measured at 16 evenly spaced concentric regions in the femoral neck using the Bone Investigational Toolkit plug‐in for the QCT Pro CTXA hip module (Fig. 2*C*). Quality assurance of the CT scanner and phantom was performed monthly according to the manufacturer's specifications.

#### Biomarkers of bone turnover

Blood samples were collected at baseline, and at 6 and 12 months via venipuncture after an overnight fast (of ≥10 hours) and abstinence from physical activity for the previous 24 hours. After centrifugation for 20 minutes at 4°C, aliquots of serum were stored at −70°C. Analyses of a bone formation marker (P1NP) and a bone resorption marker (CTx) using commercially available ELISAs as done previously^(^ ^26^ ^)^ are planned, with remaining serum stored for future use.

#### Covariate metrics

Self‐reported demographic information (ie, age, sex, race/ethnicity, education level) was assessed at baseline. Participants were queried at baseline, and at 6 and 12 months on medical information to assess 10‐year major osteoporotic and hip fracture risk using the FRAX tool (including menopausal status; glucocorticoid steroid use; fracture history; number of falls in the last year; surgery to the spine, hips, legs, and arms; diagnosis of rheumatoid arthritis; alcohol consumption; smoking status; diagnosis of type 1 diabetes mellitus; osteogenesis imperfecta in adults; untreated long‐standing hyperthyroidism; hypogonadism or premature menopause; chronic malnutrition or malabsorption; chronic liver disease; high blood calcium levels; and prior oophorectomy or hysterectomy).^(^ ^18^ ^)^ We also recorded medication use by asking participants to bring in all medications (including nutritional supplements) at the baseline assessment, and we followed up on changes in medication use that occurred at 6 and/or 12 months. Finally, height was assessed without shoes to the nearest 0.25 cm using a stadiometer (Health O Meter Portrod; Pelstar LLC, McCook, IL, USA), and body mass was measured to the nearest 0.05 kg using a calibrated and certified digital scale (Health O Meter Professional 349KLX; Pelstar LLC).

### Statistical analyses

Baseline characteristics were summarized using descriptive measures and presented overall and by treatment group as means and standard deviations (mean ± SD) for continuous variables or counts and percentages (*n* [%]) for discrete variables. Feasibility measures were compared by treatment group using independent *t* tests for continuous variables and chi‐square tests for categorical measures. Posttreatment serum calcium values were assessed by group using a general linear model and adjusting for baseline values. All statistical analyses were performed using SAS version 9.4 (SAS Institute, Cary, NC, USA) software with significance based on a type I error rate of 0.05. Overall, study feasibility was based on descriptive summary statistics of individual components rather than statistical significance.

## Results

### Baseline sample characteristics

Baseline characteristics of randomized participants are summarized overall and by group (risedronate *n* = 11; placebo *n* = 13) in Table 2. The slight imbalance in groups was caused by a miscommunication between study and pharmacy staff: One participant was provided with the wrong product. The source of the error was immediately corrected and filed as a protocol deviation, with data from the participant in question analyzed according to the product received. Average age of the study sample was 56 ± 7 years, 83% of the study sample were female (63% were postmenopausal), and 21% were black. Baseline BMI was 44.7 ± 6.3 kg/m^2^, with the risedronate group having a significantly higher BMI compared with the placebo group (48.1 ± 7.2 kg/m^2^ versus 41.9 ± 3.8 kg/m^2^, respectively). Eighty‐three percent completed schooling beyond high school. FRAX‐estimated probability of a fracture in the next 10 years was low (6.0% risk of a major fracture and 0.4% risk of a hip fracture); however, three individuals (12.5%; all in the risedronate group) were classified as osteopenic based on regional DXA assessment and the World Health Organization classification guidelines.^(^ ^27^ ^)^ Finally, average serum calcium and creatinine values were within the normal range and balanced between groups. Twenty‐two (92%) participants had eGFR >60 mL/min per 1.73 m^2^, and all were >50 mL/min per 1.73 m^2^.

**Table 2:** Baseline Characteristics of Study Sample, Overall, and by Treatment Group

Variable	Overall *n* = 24	Risedronate *n* = 11	Placebo *n* = 13
Age (y)	55.7 ± 6.7	53.8 ± 7.7	57.3 ± 5.7
Female, *n* (%)	20.0 (83.0)	9.0 (81.8)	11.0 (84.6)
Postmenopausal status, *n* (%)	15.0 (62.5)	6.0 (54.5)	9.0 (69.2)
Black, *n* (%)	5.0 (20.8)	3.0 (27.3)	2.0 (15.4)
Weight (kg)	122.1 ± 22.6	132.9 ± 25.3	113.0 ± 15.7
BMI (kg/m^2^)	44.7 ± 6.3	48.1 ± 7.2	41.9 ± 3.8
Education, *n* (%)			
High school degree or less	4.0 (16.7)	3.0 (27.3)	1.0 (7.7)
Some college	12.0 (50.0)	4.0 (36.4)	8.0 (61.5)
College+	8.0 (33.3)	4.0 (36.4)	4.0 (30.8)
FRAX 10‐year probability			
Major fracture (%)	6.1 ± 5.9	6.2 ± 7.9	5.9 ± 3.8
Hip fracture (%)	0.4 ± 0.6	0.5 ± 0.9	0.3 ± 0.3
Clinical bone categorization, *n* (%)			
Normal	21.0 (87.5)	8.0 (72.7)	13.0 (100.0)
Osteopenic	3.0 (12.5)	3 (27.3)	0 (0)
Calcium (mg/dL)	9.4 ± 0.43	9.5 ± 0.4	9.3 ± 0.3
Creatinine (mg/dL)	0.83 ± 0.17	0.85 ± 0.20	0.81 ± 0.15
eGFR (>60 mL/min per 1.73m^2^), *n* (%)	22 (92)	10 (91)	12 (92)

Continuous data are presented as mean ± SD. Categorical variables are presented as *n* (%).

eGFR = Estimated glomerular filtration rate; FRAX = Fracture Risk Assessment Tool;.

### Study feasibility

**Fig 3:**
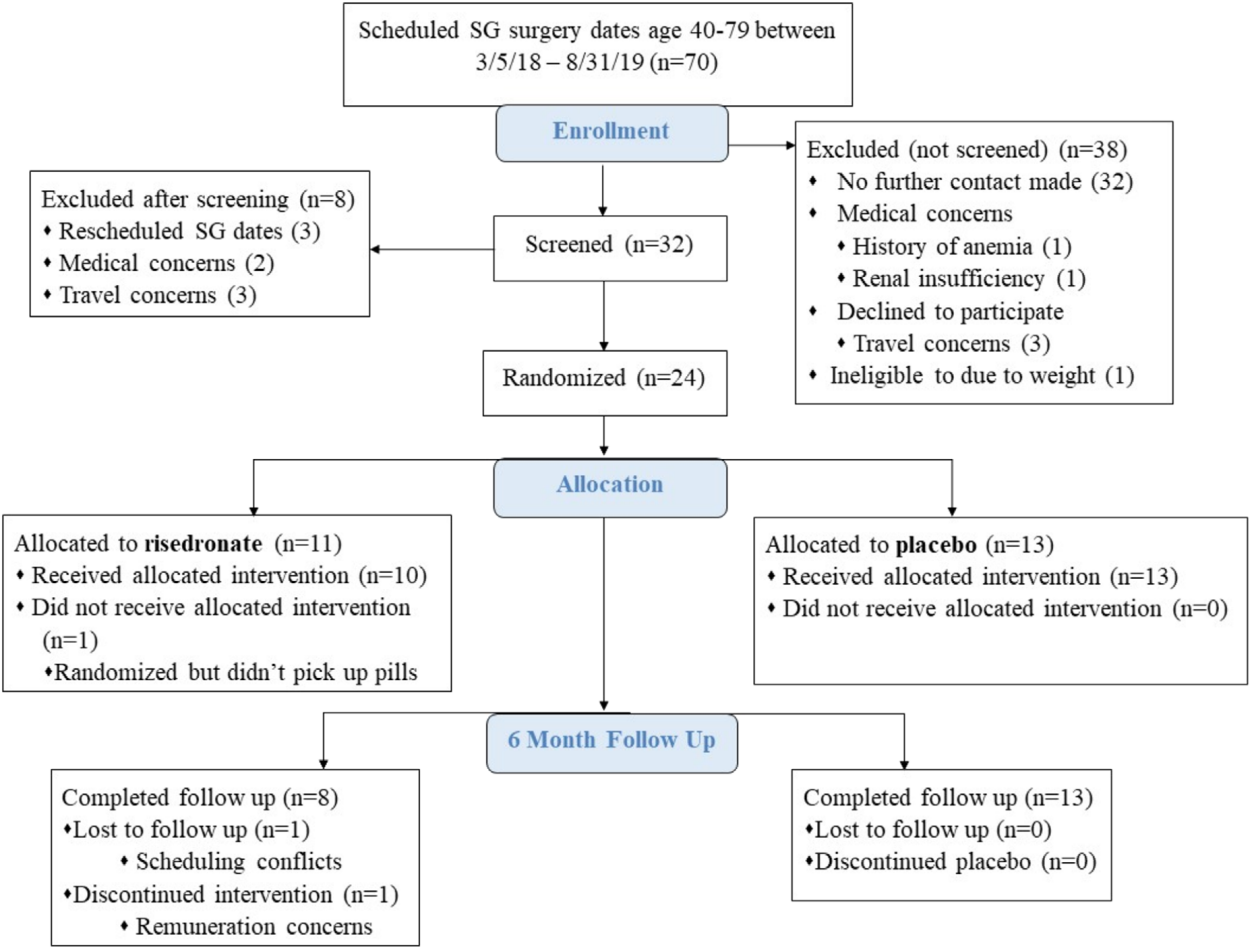
Weight Loss With Risedronate for Bone Health study CONSORT (Consolidated Standards of Reporting Trials) diagram. SG = Sleeve gastrectomy.

Seventy patients who were scheduled to receive a SG were referred to study staff for possible inclusion during the study recruitment period (March 5, 2018–August 31, 2019). Of those, 38 were excluded prior to phone screening based on further contact being made (*n* = 32), medical concerns (*n* = 2), travel concerns (*n* = 3), and weight ineligibility (*n* = 1). After phone screening, eight were excluded because of rescheduled SG dates (*n* = 3), medical concerns (*n* = 2), and travel concerns (*n* = 3). The remaining 24 (34% yield) were randomized to risedronate (*n* = 11) or placebo (*n* = 13). Twenty‐one participants completed all 6‐month follow‐up requirements (retention, *n* = 88%) with all (*n* = 3) loss to follow‐up occurring in the risedronate group (see Fig. 3). Reasons for loss to follow‐up included a scheduling conflict, concern about remuneration, and inability to pick‐up medication dosages prior to the surgery date. Importantly, no participant withdrawals were based on intolerance of the study drug.

Complete information on feasibility metrics are presented in Table 3. Among all randomized participants, the average number of pills taken was 5.5 ± 1.4. After removing participants who withdrew or were lost to follow‐up, the average number of pills taken increased to 5.9 ± 0.4 and 6.0 ± 0.0 in the risedronate and placebo groups, respectively (*p* = 0.21). Ninety‐two percent of randomized participants took >80% of pills, which increased to 100% after excluding those who withdrew. Five AEs (out of 134 contacts; 3.7% AE rate) were reported (see Table 4), with two occurring in the risedronate group (*n* = 11) and three occurring in the placebo group (*n* = 13; *p* = 0.84): three mild and not related, one moderate and not related, and one mild and definitely related, with one participant presenting with two AEs (second and third listing in Table 4). Posttreatment serum calcium values did not differ by treatment group (risedronate: 9.4 mg/dL; 95% CI, 9.2–9.7 versus placebo: 9.2 mg/dL; 95% CI, 9.0–9.3) and hypocalcemia (serum calcium <8.5 mg/dL) was not observed in any participant (range, 8.8–10.1 mg/dL). Finally, at their 6‐month follow‐up assessment, participants (who completed the study, *n* = 21) reported being highly satisfied (5.0 ± 0.0), with no difference between groups. Additionally, participants also reported overall satisfaction with the medication frequency, study length, communication with the study team, and ease of scheduling.

**Table 3:** Feasibility Metrics Presented Overall and by Group

Variable	All		Risedronate		Placebo		*p* Value
	N	Mean ± SD or *N* (%)	*N*	Mean ± SD or *N* (%)	*N*	Mean ± SD or *N* (%)	
Retention	24	21 (87.5)	11	8 (72.7)	13	13 (100.0)	0.044
Adherence							
Pills taken (all participants)	24	5.5 ± 1.4	11	5.0 ± 2.0	13	6.0 ± 0.0	0.091
Pills taken (completers)	21	6.0 ± 0.2	8	5.9 ± 0.4	13	6.0 ± 0.0	0.210
Safety							
Number of reported AEs per participant contacts	134	5 (3.7)	56	2 (3.6)	78	3 (3.8)	0.838
Participant satisfaction (1–5 Likert scale)							
Overall satisfaction	21	5.0 ± 0.0	8	5.0 ± 0.0	13	5.0 ± 0.0	1.0
Medication frequency	21	5.0 ± 0.0	8	5.0 ± 0.0	13	5.0 ± 0.0	1.0
Study duration	21	4.9 ± 0.2	8	5.0 ± 0.0	13	4.9 ± 0.3	0.447
Study team communication	21	5.0 ± 0.0	8	5.0 ± 0.0	13	5.0 ± 0.0	1.0
Ease of scheduling	21	5.0 ± 0.0	8	5.0 ± 0.0	13	5.0 ± 0.0	1.0

Continuous data are presented as mean ± SD. Categorical variables are presented as *n* (%).

AE = Adverse event; completers = participants who completed dosing sequence.

**Table 4:** Adverse Events Reported Within 6 Months of Intervention

Group allocation	Description	Point of occurrence (wk)	Severity	Relatedness to intervention
Placebo	Headache: blood pressure medication was forgotten	11.4	Mild	Not related
Risedronate	Scalp rash: diagnosed by dermatologist as psoriasis	9.0	Mild	Not related
Risedronate	Nausea: developed after failing to comply with medication protocol (ie, do not lie down for 30 min after taking)	13.3	Mild	Definitely related
Placebo	Exacerbation/flare of gastroesophageal reflux disease	13.4	Moderate	Not related
Placebo	Nausea: caused by acute illness (sinus drainage)	8.7	Mild	Not related

## Discussion

The main objective of the WE RISE pilot RCT was to evaluate the feasibility of using a monthly, oral bisphosphonate to mitigate surgical bone loss in patients who underwent SG. Here we reported the study design details, as well as feasibility data, assessed by participant: (i) recruitment and retention, (ii) adherence and safety, and (iii) satisfaction. Overall, we conclude that the use of risedronate in this population is feasible based upon (i) overall recruitment and retention rates of 34% and 88%, respectively; (ii) 100% pill count adherence among completers (92% when noncompleters were included) and a 3.7% AE rate (none severe, along with the absence of posttreatment hypocalcemia); and (iii) 100% of completers reporting overall study satisfaction.

To our knowledge, this is the first study designed to utilize bisphosphonate therapy as a prophylactic countermeasure to bone loss secondary to bariatric surgery. As such, we turn to separate RCT literature enrolling patients who have had bariatric surgery and assessing bisphosphonate use among older adults to provide context for our observations. Encouragingly, our recruitment rate is consistent with a recently published study aiming to enable recruitment success of patients who have had bariatric surgery (at 38%–45%); historically, recruitment yield is much lower in this population (as low as 9%).^(^ ^20^ ^)^ Attrition rates in studies of patients who have had bariatric surgery are reported to be between 3% and 63%, depending upon the type of surgery and length of follow‐up time^(^ ^28^ ^)^; thus, our 6‐month retention rate of 88% is also encouraging. Medication adherence rates are affected by dosing type and frequency; however, in general, any long‐term medication use only has an adherence rate of 50%,^(^ ^29^ ^)^ and monthly dosing of bisphosphonates falls between 47% and 53% in the first 6 months of treatment.^(^ ^30^ ^)^ The lower dosing frequency of risedronate (once monthly versus once weekly), along with its efficacy and favorable gastrointestinal profile,^(^ ^13^ ^)^ specifically guided our selection of this particular bisphosphonate. Finally, our safety results also fall in line with typical risedronate AE occurrence in nonsurgical populations (1%–8%),^(^ ^19^, ^31^ ^)^ with no serious AEs reported in our study.

Our study specifically recruited (versus other bariatric procedure) patients who have had a SG for two key reasons: (i) to minimize potential confounding by including both metabolic and restrictive surgical types, and (ii) to explicitly study what is now the most common bariatric surgery procedure. Most of the literature assessing bone health and bariatric surgery is overrepresented by patients who have had a Roux‐en‐Y gastric bypass (RYGB; historically the most utilized and well‐documented bariatric procedure).^(^ ^32^, ^33^ ^)^ In the past decade, however, over a dozen studies have reported on longitudinal changes in BMD following SG, with losses of 3% to 7% in the 6 to 12 months following surgery noted, particularly at the axial skeleton.^(^ ^2^, ^3^ ^)^ The magnitude of bone loss does appear to be less with SG than with RYGB; although losses are still considered clinically meaningful.^(^ ^3^, ^33^ ^)^ Data reporting on fracture risk specific to SG are more limited. To date, only three studies have reported on SG‐specific fracture risk.^(^ ^10^, ^11^, ^34^ ^)^ In the first two studies,^(^ ^10^, ^11^ ^)^ fracture rate/risk was similar between SG and RYGB; however, the most recent study by Paccou and colleagues suggests the risk of major osteoporotic fracture is only increased with RYGB (HR: 1.7, 95% CI: 1.5–2.0), and not SG (HR: 0.95, 95% CI: 0.79–1.14).^(^ ^34^ ^)^ Moreover, authors report that SG is actually protective against proximal humerus fractures (HR: 0.65, 95% CI: 0.45–0.94).^(^ ^34^ ^)^ Although reassuring, these findings need to be replicated before definitive conclusions can be drawn, but they do reinforce the observation that each surgical procedure carries unique fracture risk.

This novel study has several strengths worth noting. First, we capitalize on the design strengths of the RCT, including protections from known and unknown confounders and bias. Second, we increased clinical impact by specifically targeting a patient population at increased risk of bone loss caused by surgery and age. Although the use of risedronate was off‐label (ie, patients were not osteoporotic, not all were postmenopausal), the diversity within our study sample increases generalizability and suggests that we can recruit a diverse sample in a larger trial to examine subgroup differences. Third, the choice to utilize risedronate as the active intervention was intentional, and adherence and safety data are encouraging; however, it should be acknowledged that oral bisphosphonates have low bioavailability and that other antiresorptive medications (ie, zolendronate, denosumab) could be considered. Finally, inclusion of a study‐specific satisfaction questionnaire allowed the WE RISE trial to comment on several aspects of feasibility, which will collectively be used to aid in the design of a future trial. Despite these strengths, our study is not without its limitations. Most notably is the small sample and short duration of follow‐up. As the primary goal of this study was to gather preliminary knowledge and data, results certainly should not be considered definitive and were susceptible to imbalances. For example, despite randomization to treatment groups, weight/BMI was imbalanced at baseline. Additionally, though the low AE rate and lack of serious AEs is encouraging, we were not powered to adequately assess safety and certainly the loss of three participants from the risedronate group should be noted.

In sum, we conclude that results from this pilot trial suggest that the use of once‐monthly oral risedronate for 6 months in a patient population that have had a SG for the prophylactic management of surgical weight loss associated bone loss appears feasible. Forthcoming data from this study will yield information on the initial treatment effect estimates between risedronate and placebo on changes in DXA/QCT‐derived bone metrics and biomarkers of bone turnover, as well as intervention legacy effects over 12 months. As bariatric surgery—and SG in particular—is increasingly utilized to treat severe obesity and improve cardiometabolic outcomes, more data reporting on skeletal outcomes are needed. In particular, trials designed to optimize adherence to and treatment effects of pharmacologic strategies, along with those identifying other countermeasure therapies (including lifestyle‐based approaches),^(^ ^35^, ^36^, ^37^ ^)^ are needed to guide practitioners on how to best manage the skeletal consequences of bariatric surgery.

## Disclosures

The authors declared no relevant conflicts of interest.

## Author Contributions

**Ashlyn Swafford:** Data curation; formal analysis; investigation; methodology; project administration; supervision; validation; writing‐original draft; writing‐review and editing. **Jamy Ard:** Conceptualization; formal analysis; methodology; project administration; resources; supervision; validation; writing‐review and editing. **Daniel Beavers:** Data curation; formal analysis; validation; writing‐review and editing. **Peri Gearren:** Conceptualization; investigation; resources; supervision; validation; writing‐review and editing. **Adolfo Fernandez:** Conceptualization; resources; supervision; validation. **Sherri Ford:** Data curation; investigation; methodology; project administration; supervision; validation; writing‐review and editing. **Katelyn Greene:** Data curation; formal analysis; investigation; software; supervision; validation; writing‐review and editing. **Daniel Kammire:** Conceptualization; data curation; investigation; methodology; project administration. **Beverly Nesbit:** Conceptualization; investigation; methodology; project administration; resources; supervision; validation; writing‐review and editing. **Kylie Reed:** Data curation; formal analysis; investigation; software. **Ashley Weaver:** Conceptualization; data curation; formal analysis; funding acquisition; investigation; methodology; project administration; resources; supervision; validation; visualization; writing‐review and editing. **Kristen Beavers:** Conceptualization; data curation; formal analysis; funding acquisition; investigation; methodology; project administration; resources; supervision; validation; visualization; writing‐review and editing.

### Peer Review

The peer review history for this article is available at https://publons.com/publon/10.1002/jbm4.10407.

## Supporting information


**Supplementary Figure S1**. WE RISE Six Month Satisfaction Survey.

**Supplementary Figure S2**. Exemplar QCT output with phantom in view and displaying weight change in participants.
