# Supplementary figures and images for: Risedronate to Prevent Bone Loss After Sleeve Gastrectomy: Study Design and Feasibility Report of a Pilot Randomized Controlled Trial
Source: JBMR Plus. 2020 Oct 2;4(10):e10407. doi: 10.1002/jbm4.10407 (PMC7574708; doi:10.1002/jbm4.10407)

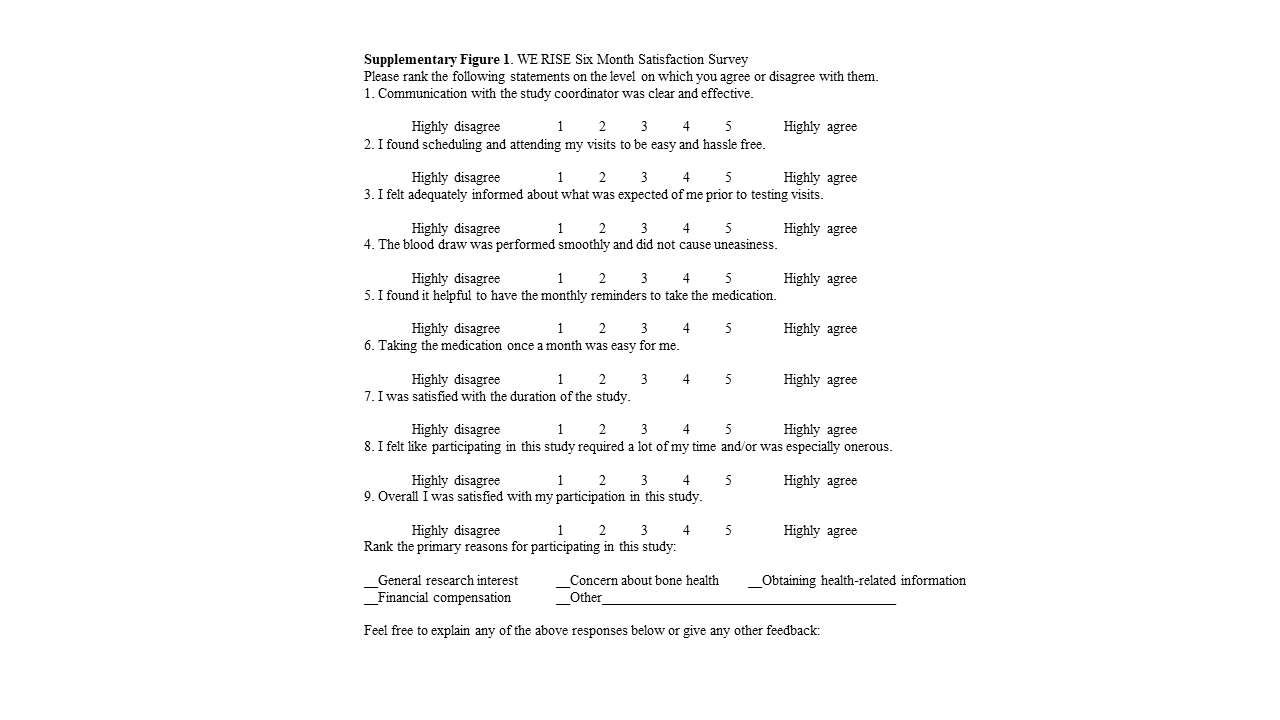

Supplement: Supplementary file 1 — Supplementary Figure S1. WE RISE Six Month Satisfaction Survey. [file JBM4-4-e10407-s001.tif]

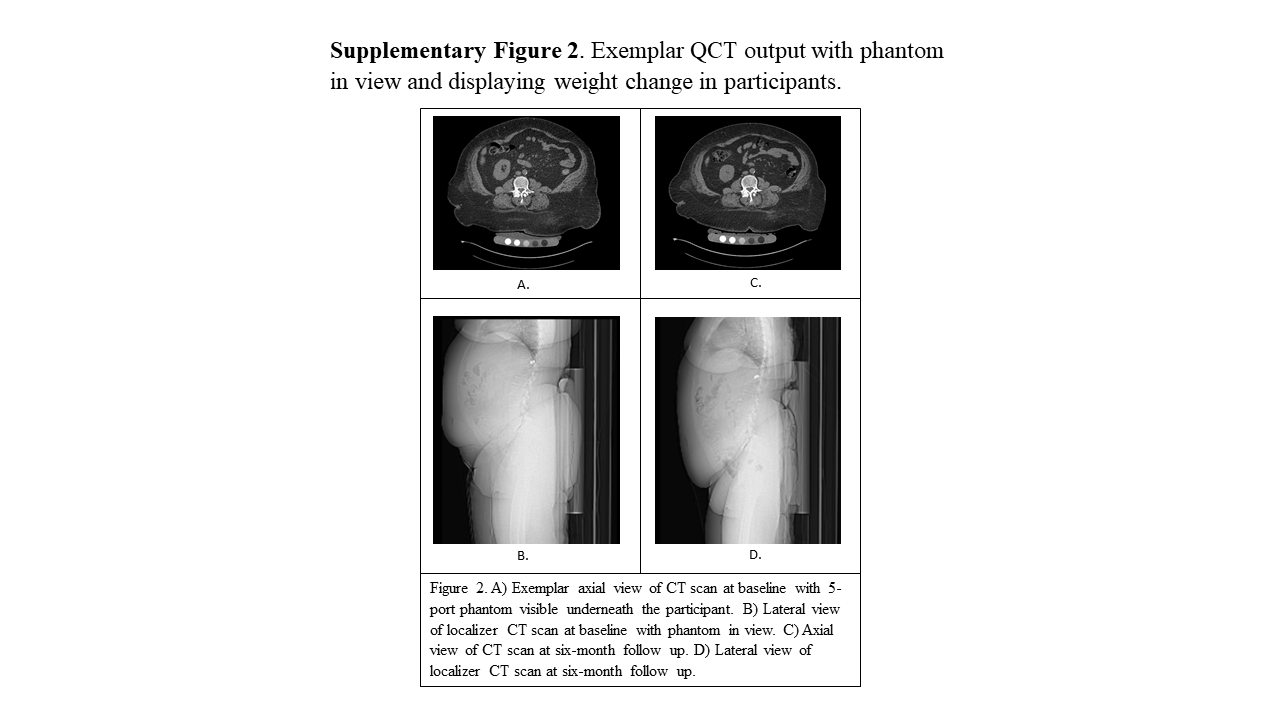

Supplement: Supplementary file 2 — Supplementary Figure S2. Exemplar QCT output with phantom in view and displaying weight change in participants. [file JBM4-4-e10407-s002.tif]
